# A nanobody-based tracer targeting DPP6 for non-invasive imaging of human pancreatic endocrine cells

**DOI:** 10.1038/s41598-017-15417-2

**Published:** 2017-11-09

**Authors:** Alexander Balhuizen, Sam Massa, Iris Mathijs, Jean-Valery Turatsinze, Jens De Vos, Stéphane Demine, Catarina Xavier, Olatz Villate, Isabelle Millard, Dominique Egrise, Carmen Capito, Raphaël Scharfmann, Pieter In’t Veld, Piero Marchetti, Serge Muyldermans, Serge Goldman, Tony Lahoutte, Luc Bouwens, Decio L. Eizirik, Nick Devoogdt

**Affiliations:** 1 ULB-Center for Diabetes Research and Welbio, Université Libre de Bruxelles (ULB), Route de Lennik 808-CP618, 1070 Brussels, Belgium; 2 Laboratory of Cellular and Molecular Immunology (CMIM), Vrije Universiteit Brussel (VUB), Brussels, Belgium; 3 In vivo Cellular and Molecular Imaging Laboratory (ICMI), Vrije Universiteit Brussel (VUB), Brussels, Belgium; 4 Cell Differentiation Laboratory, Vrije Universiteit Brussel (VUB), Brussels, Belgium; 5 Centre for Microscopy and Molecular Imaging (CMMI), Université Libre de Bruxelles (ULB), Gosselies, Belgium; 6 INSERM U1016, Université Paris-Descartes, Institut Cochin, Paris, France; 7 Department of Pathology, UZ-Brussel, Vrije Universiteit Brussel (VUB), Brussels, Belgium; 8 Department of Endocrinology and Metabolism, University of Pisa, Pisa, Italy

## Abstract

There are presently no reliable ways to quantify endocrine cell mass (ECM) *in vivo*, which prevents an accurate understanding of the progressive beta cell loss in diabetes or following islet transplantation. To address this unmet need, we coupled RNA sequencing of human pancreatic islets to a systems biology approach to identify new biomarkers of the endocrine pancreas. Dipeptidyl-Peptidase 6 (DPP6) was identified as a target whose mRNA expression is at least 25-fold higher in human pancreatic islets as compared to surrounding tissues and is not changed by proinflammatory cytokines. At the protein level, DPP6 localizes only in beta and alpha cells within the pancreas. We next generated a high-affinity camelid single-domain antibody (nanobody) targeting human DPP6. The nanobody was radiolabelled and *in vivo* SPECT/CT imaging and biodistribution studies were performed in immunodeficient mice that were either transplanted with DPP6-expressing Kelly neuroblastoma cells or insulin-producing human EndoC-βH1 cells. The human DPP6-expressing cells were clearly visualized in both models. In conclusion, we have identified a novel beta and alpha cell biomarker and developed a tracer for *in vivo* imaging of human insulin secreting cells. This provides a useful tool to non-invasively follow up intramuscularly implanted insulin secreting cells.

## Introduction

The pancreatic beta cell mass (BCM) is established around the second decade of life^1–3^, and the rate of BCM loss in patients affected by type 1 diabetes is variable^2^. There are presently no accurate ways to quantify human endocrine cell mass (ECM; the combined mass of alpha and beta cells, including active and dormant cells) and to follow up the survival of transplanted pancreatic islets *in vivo* without pre-labelling or modifications of the implanted cells^3,4^. Most work in the field focused on imaging beta cells only^5^, but beta cells can degranulate/dedifferentiate^6^ resulting in hormone negative cells^7^. Furthermore, alpha cells may be induced to transdifferentiate into beta cells^8^, emphasizing the interest of measuring the global mass of both cell types.

Limiting factors for *in vivo* endogenous ECM imaging are the low contribution (1–2%) of endocrine cells to the total pancreas mass, the fact that pancreatic islets are dispersed throughout the pancreas, and their shared embryological origin with other pancreatic cells^3^. Non-invasive *in vivo* molecular imaging of ECM thus requires a stable and highly expressed target in beta and alpha cells that can be targeted by a suitable radiotracer, and that show limited expression in exocrine cells and in extra-pancreatic tissues^3,9^. Positron-emission tomography (PET) and single-photon computed tomography (SPECT) are suitable modalities for ECM imaging, as they have high sensitivity (in the pico/nanomolar-range)^10^, a (sub)millimeter spatial resolution and proven performances in translational models with a growing number of tracers^11^.

To identify and develop novel tracers for ECM, we used a systems biology approach to mine the human pancreatic islet transcriptome for suitable islet biomarkers^12^. This approach then based on array analysis has allowed us to identify a beta cell specific biomarker, namely FXYD2γa^13^. We have now identified, based on RNA sequencing, a novel ECM biomarker that is expressed on the cell surface of pancreatic endocrine cells, namely dipeptidyl peptidase 6 (DPP6). We next developed a nanobody-based tracer targeting DPP6. Nanobodies are the variable domain derivatives of homodimeric heavy chain-only antibodies occurring naturally in camelidae. These small (13–14 kDa) polypeptides display unique features in respect to monodispersity, immunogenicity, stability, and versatility^14^; they are amenable for a wide range of radiolabeling technologies^15–17^ and have already been used for imaging purposes by SPECT or PET in both animal models of cancer^16,17^, immunity^18,19^ or atherosclerosis^20^ and in clinic^21^. We now show that they can also be used to successfully image human insulin secreting cells implanted into the muscle of immunodeficient mice, without any pre-manipulation or loading of the transplanted cells.

## Results

### Discovery of DPP6 as an ECM-enriched gene transcript

**Figure 1:**
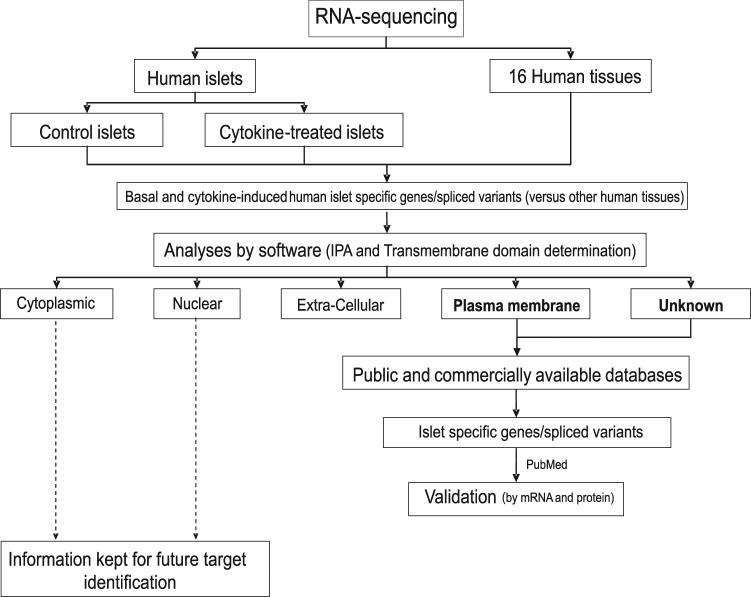
The step-by-step approach used to identify new endocrine cell biomarkers. Schematic overview of the approach taken to mine for new endocrine cell biomarkers in the transcriptome of human islet preparations (n = 5) analysed by RNA sequencing^12^ under both control condition and following treatment with pro-inflammatory cytokines (IL-1β + IFN-γ). Enriched pancreatic islet specific transcripts were identified by comparing transcriptomes of human pancreatic islets against 16 different normal human tissues. IPA: ingenuity pathway analysis, http://www.ingenuity.com/products/ipa.

**Figure 2:**
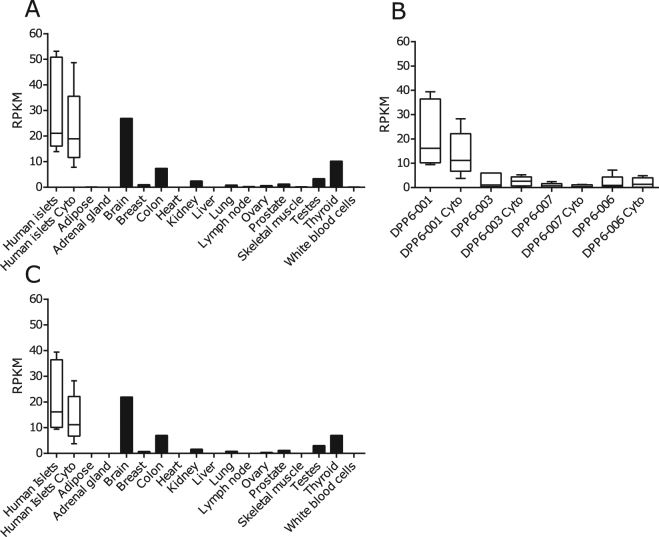
DPP6 expression in different human tissues as determined by RNA sequencing. (**A**) Expression of DPP6 mRNA based on the RNA-sequencing of human pancreatic islets; (n = 5) treated or not with IL-1β + IFN-γ (cyto) for 48 h^12^; and compared to 16 other human tissues under basal condition (Illumina Body Map 2.0; GSE30611). (**B**) Expression pattern of DPP6 splice variants in human pancreatic islets exposed or not to IL-1β + IFN-γ (cyto); human pancreatic islets express mainly the DPP6-001 variant, (n = 5); (**C**) Expression of the isoform DPP6-001 in human pancreatic islets exposed or not to IL-1β + IFN-γ (cyto), (n = 5) as compared to other human tissues; the highest extra-pancreatic expression is seen in brain, colon and thyroid.

We used a RNA sequencing-based system biology approach to identify ECM and beta cell targets^12,13^ (Fig. 1). The identification of DPP6 was based on RNA-sequenced human pancreatic islets, treated and untreated with IL-1β and IFN-γ, and on a comparison with 16 normal human tissues (ref.^12^, Illumina Body Map 2.0:GSE30611) (Fig. 2). The *DPP6* was preferentially expressed in human pancreatic islets, with a mean expression of 31 ± 8 reads per kilobase of transcript per million mapped reads (RPKM) (n = 5), several-fold higher than in other tissues, except brain (Fig. 2A). Expression of DPP6 mRNA was not modified by proinflammatory cytokines in human pancreatic islets (Fig. 2A) or by the saturated free fatty acid palmitate^22^. Furthermore, exposure of 5 human islet preparations for 24h to 28 mM glucose, as compared to 6.1 mM glucose (human preparations and experimental conditions as described in ref.^23^) did not significantly changed DPP6 expression: (qPCR corrected per actin ×10^3^), human islets at 6.1 mM glucose: 7 ± 3; human islets at 28 mM glucose: 8 ± 2 (mean ± SEM; n = 5). We have also checked expression of DPP6 in laser captured human islets obtained from type 2 diabetic patients and respective controls, as studied by microarray analysis (data from^24^). This is a more pathophysiological relevant condition, where human islets are chronically exposed to metabolic stress. The data obtained (mean ± SEM; n = 10) in respective Controls and T2D are, 539 ± 46 and 445 ± 32 (n = 10) again did not show a significant difference between groups. As a whole, the above information indicates that neither inflammation- nor metabolic-induced stress significantly modifies DPP6 expression in human islets.

DPP6-001 (ENST00000404039.5) was the main DPP6 splice variant present in human pancreatic islets (Fig. 2B), and this splice variant showed a higher expression as compared to other tissues evaluated, again except for brain (Fig. 2C). Analysis of available databases of purified human endocrine cells^25,26^ indicated that DPP6 is expressed both in beta cells (84 ± 26 transcripts per million (TPM)) and alpha cells (61 ± 20 TPM)^25^, but not in acinar or ductal cells^26^.

### Expression of DPP6

**Figure 3:**
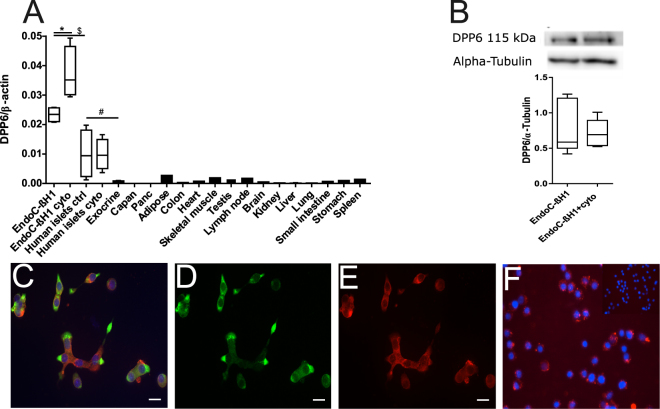
Expression of DPP6 in human islets and EndoC-βH1 cells and other tissues evaluated by qPCR and histology. (**A**) Quantitative RT-PCR (qPCR) of DPP6 mRNA expression (detecting a shared sequence among all DPP6 splice variants) in EndoC-βH1 cells (n = 5) and human pancreatic islets (n = 4) that were exposed or not to cytokines (IL-1β + IFN-γ) for 48 h, as compared to pancreatic exocrine tissue (n = 6), two exocrine cell lines (Capan-2 (n = 3) and PANC (n = 3)), and 14 other non-pathological human tissues (n = 1). (**B**) Immunoblot of EndoC-βH1 cells under control conditions or following a 48h exposure to cytokines (IL-1β and IFN-γ), with alpha-tubulin as a reference protein. A representative figure is shown at the top and densitometric analysis at the bottom (n = 5), this figure displays a cropped blot, the full-length version is included in supplementary figure 8; (**C**–**F**) Immunocytochemistry of EndoC-βH1 cells. (**C**) An overlay with cells stained with an anti-DPP6 monoclonal antibody (mAb, red), co-stained for insulin (green) and Hoechst in blue. The separate channels are displayed in (**D**) insulin (green) and (**E**) DPP6 (red) (n = 3). The mostly surface localization of DPP6 (red) can be observed in (**F**) (n = 4), with blue signals indicating Hoechst staining. The negative staining control of EndoC-βH1 cells (without the DPP6 antibody) is placed in the top right corner of panel F. White scale bar represents 1 µm. RT-qPCR and the western blot data are presented as means ± SEM. Paired and unpaired two-way ANOVA (indicated with * and $, respectively), and unpaired one-way ANOVA (indicated with #) with Šídák correction for multiple comparisons; *, $ and # p ≤ 0.05 as indicated by bars.

The RNA-sequencing data was confirmed by qPCR in 14 different human tissues as compared to human pancreatic islets and in insulin-producing EndoC-βH1 cells treated or not with cytokines (Fig. 3A), as well as human exocrine material and two exocrine cell lines Capan-2 and PANC. In the pancreatic islets DPP6 expression was nearly 20-fold higher (p ≤ 0.05) than in the exocrine material (which contained around 1–2% beta cells), while both Capan-2 and PANC cells had at least 4000 times lower DPP6 expression. There was again a higher expression of DPP6 in human pancreatic islets as compared to the surrounding extra-pancreatic tissues, and DPP6 expression was not modified by proinflammatory cytokines (Fig. 3A). The DPP6 expression was even higher in insulin-producing EndoC-βH1 cells (Fig. 3A), a finding confirmed by histology (Fig. 3C–E), which also indicated a cell surface localization of the biomarker (Fig. 3F). In these cells, the expression of DPP6 mRNA increased by 1.5-fold (p ≤ 0.05) after exposure to proinflammatory cytokines IL-1β and IFN-γ (Fig. 3A), but this increase was not observed at the protein level (Fig. 3B). The lack of cytokine-induced changes in human islets (mRNA level) or in EndoC-βH1 cells (protein level) suggests that the inflammation prevailing in T1D will not modify the expression of this biomarker.

### Detection of DPP6 by immunohistochemistry of human pancreas

**Figure 4:**
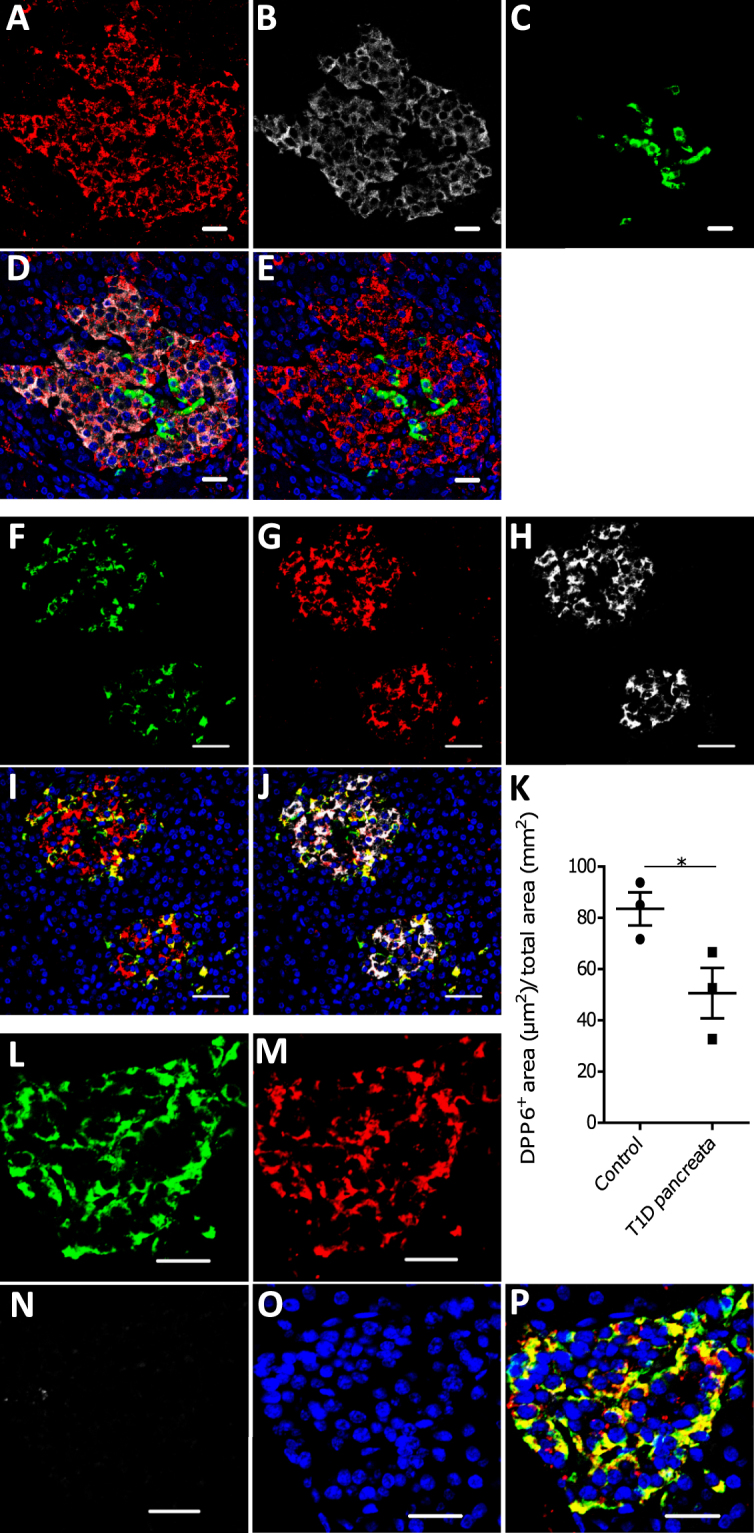
Localization of DPP6 expression in human pancreas. (**A**–**E**) A representative human pancreas stained for DPP6 (A, red), insulin (B, white), somatostatin (C, green); overlay of DPP6 (red), insulin (white) and somatostatin (green) (**D**); overlay of DPP6 (red) and somatostatin (green) (**E**); the data indicate co-staining of insulin and DPP6, but not somatostatin and DPP6; (**F**–**J**) A representative human pancreas stained for glucagon (F, green), DPP6 (G, red), insulin (H, white); DPP6 (red) and glucagon (green) overlay (**I**); overlay of DPP6 (red), insulin (white) and glucagon (green) (**J**), the data indicate co-staining of both insulin and glucagon with DPP6; (**K**) Morphometric quantification of DPP6 area in pancreata from T1D patients as compared to control, non-diabetic individuals (n = 3). (**L**–**P**) A representative human pancreas from a subject with long-term type 1 diabetes (16 years of disease) stained for glucagon (L, green), DPP6 (M, red), insulin (N, white); Hoechst (O, blue); (**P**) overlay of DPP6 (green), glucagon (red), insulin (white) and Hoechst (blue), indicating that in the absence of insulin positive cells, the remaining glucagon positive cells co-stain for DPP6. In total, 3 pancreata from normoglycemic individuals and 3 from type 1 diabetes subjects were analysed. White scale bar represents 20 µm.

The histological localization of DPP6 on normal human pancreata was investigated using a commercial mAb targeting the protein (Fig. 4). DPP6 co-localized with insulin (Fig. 4D,J) and glucagon positive cells (Fig. 4I) but not with somatostatin positive cells (Fig. 4E). DPP6 immunoreactivity was present in 90 ± 3% of insulin cells and 74 ± 10% of glucagon cells (n = 3). There were only rare cells positive for DPP6 in the exocrine pancreas, with no clear evidence for staining in acinar or duct cells (Supplementary Fig. 1). Analysis of pancreata obtained from patients with long-term type 1 diabetes (n = 3) showed nearly complete loss of insulin positive cells (Fig. 4N), while some DPP6 (Fig. 4L) and glucagon positive cells (Fig. 4M) remained. Pancreata from T1D patients had a reduced DPP6 positive area when compared to the healthy controls (p ≤ 0.05) (Fig. 4K). Interestingly, DPP6 expression was also clearly present in the insulin positive cells from two separate human insulin producing tumors (insulinomas) (Supplementary Fig. 2).

### Generation and *in vitro* characterization of nanobodies against DPP6 and selection of the lead nanobody 4hD29

**Figure 5:**
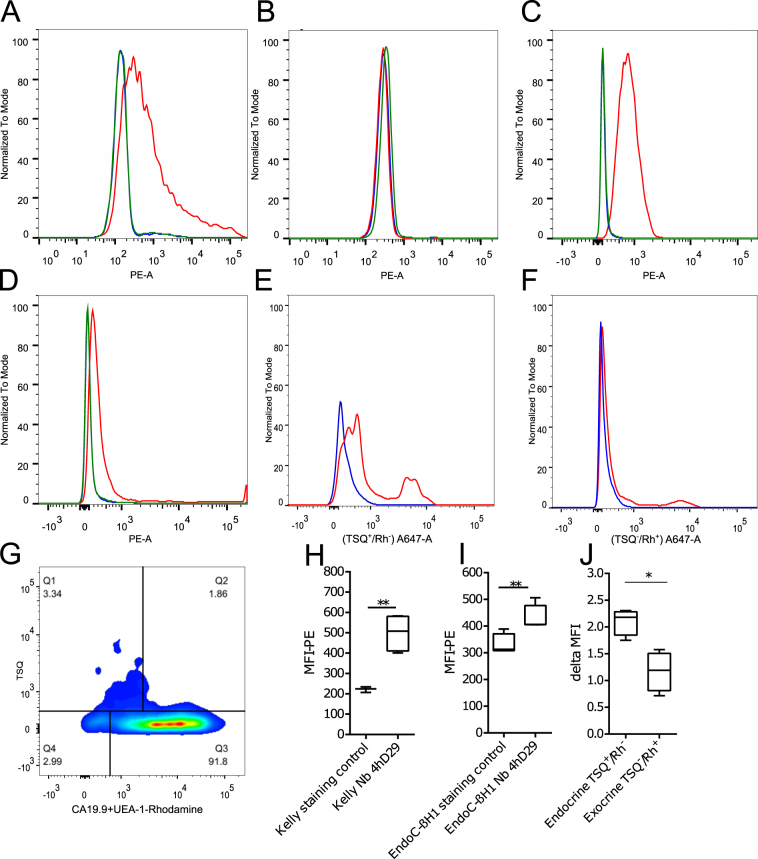
Flow cytometry analysis of nanobody cell binding. The cell binding of the 4hD29 nanobody (Nb) was evaluated by flow cytometry. (**A**–**D**) 4hD29 (red) recognizes human DPP6 in different cell types (HsDPP6); (**A**) 4hD29 (red) labelled transiently-transfected CHO cells overexpressing DPP6, where neither the irrelevant control Nb (green) nor the secondary control antibodies only (blue) stained the cells (n = 3); (**B**) non-transfected CHO cells labelled as in A (n = 3); (**C**) The Nb 4hD29 (red) recognizes DPP6-positive human Kelly neuroblastoma (n = 4) and (**D**) EndoC-βH1 cells (n = 5), whereas the secondary antibodies only (blue) or an irrelevant control Nb (green) do not. (**E**,**F**) 4hD29 (red) binds to endocrine (TSQ^+^/Rh^−^) (**E**), but not to exocrine tissue (TSQ^−^/Rh^+^) (**F**) of dissociated human pancreas (n = 4). Background staining with secondary staining control is indicated in blue. (**G**) Overview of the gating strategy for endocrine (TSQ^+^/Rh^−^) and exocrine cells (TSQ^−^/Rh^+^) analysed in (**E**,**F**). The median fluorescence intensity (MFI) was calculated for Kelly neuroblastoma (**H**) and EndoC-βH1 cells (**I**). Delta MFI values were calculated to compare the endocrine (TSQ^+^/Rh^−^) and exocrine populations (TSQ^−^/Rh^+^), showing that 4hD29 has an increased binding in endocrine cells as compared to exocrine cells (**J**). Unpaired (**E**,**F**) or paired (**J**) Student’s *t*-tests were performed to compare two groups; *p ≤ 0.05, **p ≤ 0.01.

Anti-DPP6 nanobodies were generated by immunization of a dromedary with a recombinant protein, derived from the extracellular domain of the HsDPP6 transmembrane protein. Biopannings were subsequently performed of a phage-displayed immune nanobody library on the recombinant protein, and selection of binders by phage-ELISA. Thirteen nanobodies with different protein sequences were identified as binding to HsDPP6 recombinant protein and divided into 9 families based on the homology of their complementarity determining region (CDR) 3. Among these nanobodies (Supplementary Fig. 3J) eight were successfully produced and purified as hexahistidine-tagged proteins from *E*. *coli* fermentation cultures and the nanobodies’ affinities were analysed using Surface Plasmon Resonance (SPR) (Supplementary Fig. 3A–H). The best scoring nanobody, designated 4hD29, had an affinity (K_D_ value) towards DPP6 of 1.2 nmol/l (Supplementary Fig. 3H,I). Subsequently, the 8 nanobodies were screened for their recognition of the transmembrane DPP6 on cells using flow cytometry. Nanobody 4hD29 (Fig. 5A) and 5 other nanobodies (Supplementary Fig. 4), but not the control non-specific nanobody, showed clear binding to HsDPP6 transiently-transfected CHO cells (Fig. 5A). None of these nanobodies bound to wild type CHO cells (Fig. 5B), except for nanobody 3hD36 that also non-specifically stained untransfected cells (data not shown). Despite a 93% homology between human and murine DPP6 in their extracellular region, 4hD29 did not recognized murine DPP6 in transiently-transfected CHO cells (data not shown). Next, we evaluated the nanobody candidates using the human Kelly neuroblastoma cell line, which expresses DPP6^27^. 4hD29 and 6 other nanobodies, but not the control non-specific nanobody, bound to Kelly cells (p ≤ 0.01; n = 4 for 4hD29) (Fig. 5C,H, Supplementary Fig. 5).

Based on these flow cytometry experiments and the SPR data, nanobody 4hD29 was selected as the lead compound and its binding was further characterized on EndoC-βH1 cells where 4hD29 binding could be determined (p ≤ 0.01; n = 5) (Fig. 5D,I). Finally, 4hD29 bound significantly (p ≤ 0.05; n = 4) to human endocrine tissue (Fig. 5E,J) as compared to exocrine tissue (Fig. 5F,J), where no 4hD29 binding could be observed. Endo- and exocrine tissue was differentially gated as shown in Fig. 5G.

### Biodistribution and *in vivo* imaging of mice xenografted with human cells expressing DPP6

**Figure 6:**
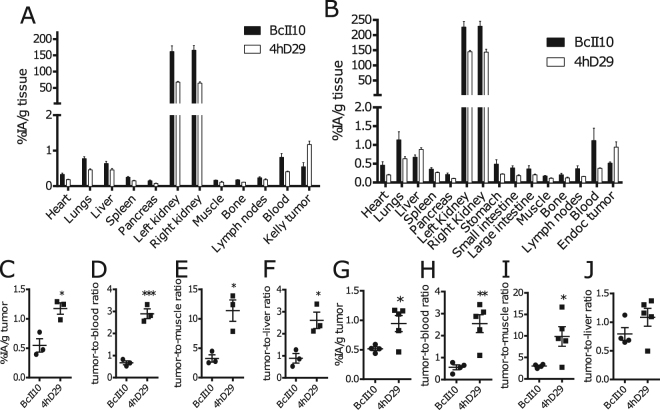
*Ex vivo* biodistribution profile of radiolabelled 4hD29 and control nanobodies in mice implanted with a Kelly neuroblastoma subcutaneous tumour or intramuscular EndoC-βH1 transplants. *Ex vivo* biodistribution analysis of radiolabelled 4hD29 or control (non-specific) nanobody (BcII10) was performed in mice xenografted with Kelly neuroblastoma cells (n = 4) (**A**,**C**–**E**) or with EndoC-βH1 transplants (n = 5) (**B**,**F**–**H**). The evaluation was done 60 or 80 minutes, respectively, after i.v. administration of 5 µg ^99m^Tc-labelled nanobody 4hD29 (white bars) or control nanobody BcII10 (black bars) and expressed as percent of injected activity per gram of tissue (%IA/g) ± SEM; (**C**,**F**) Individual uptake levels of tracers in Kelly tumours (**C**) or EndoC-βH1 transplants (**F**). Tumour-to-blood (**D**,**G**) and tumour-to-muscle ratios (**E**,**H**) of individual mice with either Kelly tumours (**D**,**E**) or EndoC-βH1 transplants (**G**,**H**). Data is presented as mean ± SEM; unpaired *t*-test, *p ≤ 0.05; **p ≤ 0.01; ***p ≤ 0.001.

**Figure 7:**
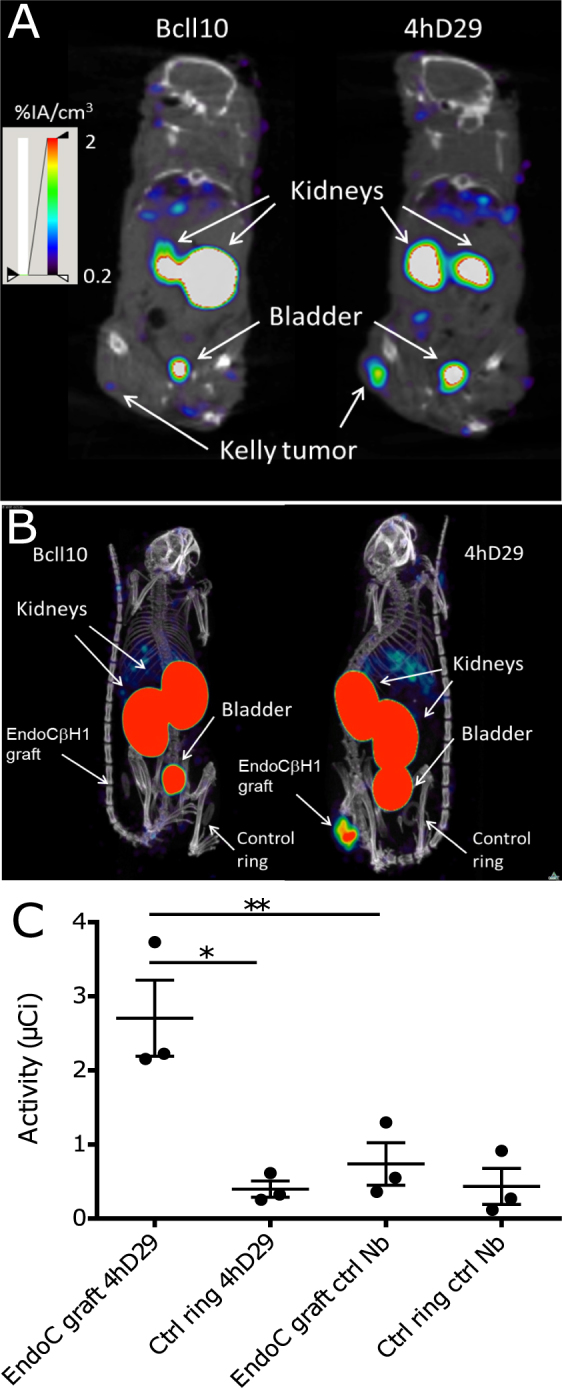
Noninvasive microSPECT/CT imaging of Kelly neuroblastoma and EndoC-βH1 tumours in mice using 4hD29 or control nanobody tracers. Representative microSPECT/CT fusion images of mice bearing subcutaneous human Kelly neuroblastoma tumours (n = 3 per Nanobody); coronal slices at the height of the tumour are shown) (**A**) or EndoC-βH1 transplants (n = 5–6 per Nb; maximal intensity projections are shown) (**B**). The images were obtained at 60 and 80 minutes, respectively, after i.v. administration of 5 µg of ^99m^Tc-4hD29 (right panel) or ^99m^Tc-BcII10 (left panel) radiotracers. Mice were implanted with EndoC-βH1 grafts in transplantation rings in the right flank and empty transplantation rings in the left flank. (**C**) Quantification of SPECT signals in equally-sized regions-of-interest (ROIs) drawn over each transplantation ring in each individual mouse. Data are expressed as mean of total radioactivity (µCi) in each ROI ± SEM; Paired two-way ANOVA with Šídák correction for multiple comparisons, *p ≤ 0.05 and **p ≤ 0.01 as indicated by bars.

We next investigated whether the lead nanobody 4hD29 can be used as an *in vivo* imaging diagnostic tool to monitor DPP6-expressing cells. For this purpose, two humanized mouse models were used, namely immunodeficient mice with s.c. Kelly tumours grown for about 2-weeks or implanted intramuscularly with EndoC-βH1 cells in rubber rings (empty rings were used as negative controls; see Methods). Implanted EndoC-βH1 cells grew slowly, and only generated palpable tumours after 9–10 weeks, while the mice had a normal growth (Supplementary Fig. 6A,D). At this stage, the mice became hypoglycemic, which was partially counteracted by addition of 20% glucose in the drinking water (Supplementary Fig. 6B,E) and high levels of human C-peptide were detectable in serum samples (Supplementary Fig. 6C,F). Using these models, microSPECT/CT imaging and *ex vivo* biodistribution analyses were done respectively 60 and 80 minutes after i.v. administration of ^99m^Tc-labelled nanobody 4hD29 or negative control nanobody BcII10 (Figs 6 and 7).

Based on the biodistribution analyses (Fig. 6A,B) and the images (Fig. 7) in both mouse models, ^99m^Tc-4hD29 displayed a 2-fold higher uptake over ^99m^Tc-BcII10 in the Kelly neuroblastoma tumour (1.2 ± 0.10 %IA/g) vs. (0.5 ± 0.1 %IA/g) (Fig. 6C) and in the EndoC-βH1 tumour (1.0 ± 0.1 %IA/g) vs. (0.5 ± 0.04 %IA/g) (Fig. 6G). Both radiolabelled 4hD29 and control nanobodies showed a high uptake in the kidneys, bladder and a fast wash-out from blood and other non-targeted tissues. Tumour 4hD29 radioactivity levels were specific, since tumour-to-blood and tumour-to-muscle ratios were respectively 2.9 ± 0.2 and 9.9 ± 3.2 in neuroblastoma-bearing mice (Fig. 6D,E) and 2.5 ± 0.4 and 9.9 ± 2.2 in EndoC-βH1-bearing mice (Fig. 6H,I), values that were respectively 4.3-, 3.1-, 4.6- and 3.3-fold higher than those observed for the control nanobody. Tumour-to-liver ratios were 2.6 ± 0.6 for Kelly tumours (Fig. 6F) and 1.1 ± 0.3 for the EndoC-βH1 grafts (Fig. 6J). Importantly the high tumour-to-background levels of the lead anti-DPP6 nanobody in the dissection analyses were translated into a clear visualization of tumours in fused SPECT/CT images (Fig. 7 and Supplementary video 1). Indeed, *in vivo* administration of ^99m^Tc-4hD29 (but not ^99m^Tc-BcII10) allowed visualization of both the Kelly tumour (Fig. 7A) and the EndoC-βH1 transplant (Fig. 7B and Supplementary videos 1 and 2) with high contrast. Image quantification in the latter mice (Fig. 7C) showed a 6.8-fold higher signal of ^99m^Tc-4hD29 in the EndoC-βH1 transplant as compared to the empty vehicle ring and a 3.7-fold higher signal than that observed in the EndoC-βH1 transplant following injection of the radiolabelled control nanobody. Similar results were obtained with ^111^In-labelled nanobodies in the Kelly-tumour mouse model (Supplementary Fig. 7), where *ex vivo* quantification showed that ^111^In-4hD29 specifically accumulated in the Kelly tumour while unbound tracer was quickly removed from non-targeted tissues through renal clearance. This resulted in tumour-to-blood and tumour-to-muscle ratios as high as 16.7 and 29.1, respectively, 2h after i.v. administration.

## Discussion

We presently identified DPP6 as a new biomarker of the endocrine pancreas, expressed preferentially in human beta and alpha cells and also in insulinomas. To validate its use for *in vivo* imaging, we generated a high-affinity camelid single-domain antibody (nanobody) targeting human DPP6. Nanobodies are suitable for clinical *in vivo* imaging with nuclear modalities, both in animal models^15–17^ and clinically^21^. The lead nanobody, 4hD29, was radiolabelled and *in vivo* SPECT/CT imaging and biodistribution studies were performed in immunodeficient mice transplanted with DPP6-expressing Kelly neuroblastoma cells or insulin-producing human EndoC-βH1 cells. To our knowledge, this is the first description of the use of nanobodies for *in vivo* imaging of human insulin secreting cells. Human DPP6-expressing cells were clearly visualized in both models, with very low background.

The available knowledge of beta and alpha cell mass is based on autopsy data and it is presently not possible to follow the survival of transplanted human pancreatic islets or endogenous ECM changes over time in diabetic patients^3^. Development of both beta cell specific^5^ and ECM tracers^4^ are of importance considering the potential plasticity of mature pancreatic cells^8,28–31^ and the possibility that alpha-cells may provide a reservoir for beta-cells^8^. Furthermore, beta and alpha cells are the most frequent cell types in human pancreatic islets, and novel ECM imaging modalities would be very useful for the follow up of transplanted pancreatic islets^32^. In this context, it is important to identify targets that are not affected by inflammatory or metabolic stress, which is the case of DPP6, allowing the estimation of beta cell mass unaffected by the stressful conditions to which islet cells are exposed in diabetes or following transplantation.

Previous strategies to screen for beta cell or islet cell biomarkers have often relied on antibody arrays^13,33–35^. Our present approach to search for novel biomarkers based on RNA sequencing and analysis of the islet transcriptome, as compared to other tissues, provides a more sensitive and specific approach, avoiding the unspecific binding issue associated with antibody-based screenings^36^.

The DPP6 gene encodes for a single pass transmembrane peptidase without peptidase activity. At the protein level, DPP6 co-ensembles with Kv4-potassium channels that take part in exocytosis. DPP6 functions as a regulator of the channels’ recovery rate and voltage dependency^37^ and promotes the cell surface localization of KCND2^38^, besides other functions that remain to be identified^39^. The different splicing variants of DPP6 have different lengths in their intracellular part, with a relatively constant extracellular part^38^. The splicing variant DPP6-001 is the DPP6 variant with the highest expression in pancreatic islets (present data). All the main DPP6 variants are expressed in the central nervous system, except DPP6-001, which is preferentially expressed in pancreas^37^. Considering the expression pattern of DPP6 and the unchanged nature of the extracellular part of the full gene, we have chosen the protein encoded by the full gene as the best target, instead of only the DPP6-001 variant.

Once a relevant islet biomarker is identified, the next step is to develop ligands and tracers for *in vivo* imaging. For this purpose, we have presently generated anti-DPP6 nanobodies. The camelid nanobody tracer backbone was chosen for its inert properties, fast clearance rate and short blood retention time^17^. Antibody based approaches are less optimal in this context, as they have high non-specific retention due to their slow clearance rate^40^. On the other hand, nanobodies, due to their small size (15kDa), are cleared as small hydrophilic proteins and are excreted via the renal route^41^.

After dromedary immunization, biopanning of a phage displayed nanobody library and detailed *in vitro* characterization, the lead anti-DPP6 nanobody 4hD29 was selected for *in vivo* evaluation. 4hD29 is specific for the extracellular part of *human* DPP6, without binding to endogenous murine DPP6. Due to the species-specific nature of the nanobody, we performed all *in vivo* validation steps in humanized mouse models, i.e. mice implanted either with insulin-producing EndoC-βH1 cells in the quadriceps or femoris muscle, or xenografted in the subcutaneous tissue with the neuronal Kelly cancer line that spontaneously expresses DPP6. It will be important to confirm these findings in future experiments using implanted human islets. The tracers displayed optimal *in vivo* specificity, with high EndoC-βH1 transplant-to-muscle and high Kelly tumour-to-blood ratios, and excellent *in vivo* visualization of these DPP6-expressing cells implanted either in the muscle or in the subcutaneous tissue (Fig. 7, Supplementary Videos 1 and 2). Future experiments must test whether 4hD29 allows visualization of human islets implanted in rodents in the intraportal system, spleen, kidney capsule, peritoneum, muscle or in the subcutaneous region. Intramuscular grafting of pancreatic islets is under development^42^, while 4hD29-mediated visualization of islets implanted under the kidney capsule, and to a certain extent also of liver islet transplants, is deemed less optimal due to the potential nonspecific accumulation of nanobody-tracers at this site(s).

In conclusion, we have presently identified DPP6 as a new potential islet biomarker and generated a novel nanobody tracer that targets it. The tracer displays high specificity for DPP6 and its *in vivo* properties were validated in mice xenografted with either human insulin secreting cells or a neuronal derived tumour that spontaneously expresses DPP6.

## Methods

### Ethical statements

Pancreases not suitable for clinical purposes were obtained via the Endocrinology and Metabolism of Organ and Cellular Transplantation unit at Cisanello University Hospital at the University of Pisa, Italy and from the department of Pathology, UZ-Brussel, Vrije Universiteit Brussel (VUB), Brussels, Belgium with informed written consent and processed with the approval of the local Medical and Health Research Ethics Committees of the Pisa University, Italy and of the VUB, Belgium. The donors were anonymised, and all experiments and methods using human pancreatic islets were approved by and performed in accordance with the guidelines and regulations made by the regional Medical and Health Research Ethics committees of the Pisa University, Italy and of the VUB, Belgium. Human islet isolation and culture was performed as previously described^43^. No organs/tissues were procured from prisoners. The human pancreatic islet or pancreas preparations used in the present study are described in Supplementary Table 1. Historical material from two insulinomas was provided by the Pathology Department of the VUB. Information on the patients and tumours are provided in the legend for Supplementary Figure 2. Male NMRI-*Foxn1* ^nu^ */Foxn1* ^nu^, female SCID CB-17/Icr-*Prkdc* ^scid^/Rj mice (Janvier labs), all 6 weeks old, and C57Bl6 mice (both from Charles River Laboratories, Saint-Germain-sur-l′Arbresle, France) were used and housed according to the rules of the Belgian regulations for animal care and in accordance with the Animal Act 1986; 2013, and all experiments and methods involving these animals were performed with approval by the Ethical Committee for Animal Welfare (CEBEA) of the Medical Faculty of the ULB and ULB-Centre for Microscopy and Molecular Imaging (CMMI) (Campus Biopole Charleroi) (ethical permits CMMI-2013-03;2014 and 485N), Belgium.

### Cell culture, cell transfection and cytokine exposure

Chinese Hamster Ovary (CHO), human Kelly (pNB-1) neuroblastoma (#92110411) (both from ECACC, Salisbury, UK), pancreatic ductal adenocarcinoma (Capan-2) and epithelioid carcinoma (Panc-1) cells were cultured at 37 °C, 5% CO_2_ in Ham’s F-12 Nutrient mix (CHO), RPMI 1640 media (pNB-1 and Capan-2) or DMEM (Panc-1), supplemented with 2 mM GlutaMAX and 10% FBS. CHO cells were transfected for 72h with a pCMV6 vector containing human DPP6 variant 1 (#RC216919), or the murine homologue (#MR210746, both from Origene). The transfection was performed using Fugene HD transfection reagent per manufacturer’s instructions. Human insulin-producing EndoC-βH1 cells^44^ and human pancreatic islets were cultured as described^45^. To determine whether the expression of DPP6 is affected by inflammatory mediators, EndoC-βH1 cells and human pancreatic islets were exposed to human IL-1β and human IFN-γ for 48h^12^.

### Immunohistochemistry

For immunofluorescence cytochemistry of EndoC-βH1 cells the cells were fixed with 4% paraformaldehyde and permeabilized with 0.2% Tween-20, and thereafter incubated for 1 h with guinea pig anti-insulin mAb (1:500; #A0564) (DAKO) and mouse anti-DPP6 mAb (1:250; #MAB2360) (R&D systems). The EndoC-βH1 cells were also stained for 15 min at 4 °C with the same mAb (1:20; #MAB2360) without any prior fixation or permeabilization. Appropriate anti-guinea pig or anti-mouse Alexa fluor fluorescent conjugated secondary antibodies (ThermoFisher Scientific) were applied and the slides were imaged as described^45^.

Paraffin embedded control (i.e. normoglycemic) and type 1 diabetic human pancreata were processed and stained with mouse anti-DPP6 mAb (1:20; #MAB2360) (R&D systems), and stained with anti-insulin, anti-glucagon and anti-somatostatin monoclonal antibodies (mAbs) and imaged as described^46^. The morphometrical quantification was calculated as follows: DPP6 positive area × 1000 (µm^2^) divided by the total surface area (mm^2^).

### mRNA expression

mRNA extraction, reverse transcription and quantitative PCR (qPCR) were performed as described^45^. The qPCR was directed to a sequence that amplifies all known splice variants of DPP6. cDNA from 14 human normal tissues were obtained from BioChain (San Francisco, CA, USA). The primers used were: *Homo sapiens* (Hs) *HsDPP6* forward: 5′-ACAGTGAGACTGTGGAATGTTGA-3′, reverse: 5′-GAGGATCCCCATGAGGAATTTTG-3′, (199bp); *hsACTB* forward: 5′-GCCTGGAGAAACCTGCCAAGTATGA-3′, reverse: 5′-AACCTGGTCCTCAGTGTAGCCC-3′ (101bp).

### Western blot

Immunoblot analyses was performed as in^45^ with a DPP6 mAb (1:1000, #ab198506; Abcam); alpha-tubulin mAb (1:5000; #T5168; Sigma-Aldrich) was used as a reference protein. The detection was performed with anti-rabbit or anti-mouse pAb-HRP (1:10000) (#715-036-150 and #711-036-152, Jackson ImmunoResearch Laboratories). Densitometric analyses were performed with Image Studio Lite v5.0 (Li-Cor Biotechnology).

### Mouse models hosting human cells

Male NMRI-*Foxn1* ^nu^ */Foxn1* ^nu^ and female SCID CB-17/Icr-*Prkdc* ^scid^/Rj mice were transplanted with human insulin-producing EndoC-βH1 cells as described^44,47^. Male NMRI-*Foxn1* ^nu^ */Foxn1* ^nu^ mice were used for methodological development/validation, but all follow up experiments were performed in female SCID CB-17 mice. At the day of inoculation, 4–6 × 10^6^ EndoC-βH1 cells were seeded on a rubber toric joint (EFJM), supported in Matrigel HC (Corning) supplemented with MmVEGF-164 (1 ng/ml) (BioLegend). The cell-containing or the empty vehicle rubber rings were then inserted under the epimysium in the biceps or quadriceps femoris muscle. The mice were anesthetized with 3 % isoflurane. They received short-term analgesic (Buprenorphine 0.1 mg/kg) and long-term analgesic (3 mg/ml of Acetaminophen-supplemented water for 10 consecutive days) respectively before and after the surgery. Random glycaemia was measured weekly with an ACCU-CHEK Nano glucometer (ROCHE). Once the tumour became palpable, the mice received 20 % glucose-supplemented drinking water to counter the progressive hypoglycemia induced by the EndoC-βH1 cells. Human C-peptide was measured in plasma with a human Ultrasensitive C-peptide ELISA (Mercodia). Kelly tumour-bearing mice were generated by s.c. injections of 5 × 10^6^ cells mixed 1:1 with Matrigel (Corning) in the hind leg of female Crl:NU(NCr)-Foxn1^nu^ mice.

### Generation, production and selection of Nanobodies

Nanobodies were generated as detailed elsewhere^20,40^. A recombinant protein produced in NS0 cells encoding the extracellular domain amino acids 118-865 of human DPP6 (HsDPP6, R&D Systems, Abingdon, UK) was used to immunize a dromedary, for enrichment by biopanning of a phage-displayed immune nanobody library and for screening binders by ELISA. Specific nanobody binders were sequenced and unique sequences were identified using Geneious software (Biomatters). Hexahistidine-tagged nanobodies, cloned in the pMES4 plasmid, were produced and purified from *E*. *coli* cells as previously described^40^. As a non-targeting negative control nanobody BcII10, directed against a bacterial enzyme^48^, was used. Affinity measurements were performed on a Biacore T200 instrument^40^. Briefly, recombinant DPP6 protein was immobilized on a CM5 chip using EDC/NHS chemistry. Thereafter the binding and dissociation kinetics of 9 different dilutions (2–500 nmol/l) of nanobodies were monitored as a function of time. Sensograms were analysed and curves were fitted with a 1:1 binding model to calculate equilibrium dissociation constants (K_on_, K_off_, and K_D_). Screening of nanobody candidates (labelled with Phycoerythrin (PE) mAb (1:100; #562027, BD Biosciences) for binding to the DPP6 transmembrane protein was performed by flow cytometry^40^ on DPP6 expressing cells, i.e. CHO-transiently transfected cells, EndoC-βH1 cells and Kelly neuroblastoma cells. The same approach was used to evaluate the lead nanobody, namely 4hD29 (labelled with an anti-hexahistidine secondary antibody, an anti-rabbit conjugated Alexa 647 pAb (1:500; #A32733) or with a Cy5-conjugated pAb (1:500; #A10523), both from ThermoFisher), for its binding to human pancreatic tissue. For this purpose, 10^5^ cells from crude digested human pancreata were processed and labelled as in^17,49^, with TSQ (50 µg/ml; #ENZ-52153, Enzo Life Sciences) that selectively binds islet cell granules. In parallel, the exocrine material (acinar and ductal cells) was labelled with UEA-1-Rhodamine (1:2000; #RL-1062, Vector Laboratories) to mark acinar cells; to stain the ductal cells an mAb for CA19-9 (1:100; #15146, Abcam) was used, with subsequent secondary anti-mouse pAb Rhodamine Red X (1:500; #R6393, ThermoFisher). The FACS gating strategy to separate endocrine and exocrine pancreatic tissues is shown in Fig. 5G. Acquisition was performed on a Fortezza or on a CantoII (BD biosciences) flow cytometers. The data were subsequently analysed with FlowJo 10.07 (FlowJo, LLC) and median fluorescence intensity (MFI) was calculated for PE-A and delta median fluorescence intensity (deltaMFI) (fold difference when comparing staining with 4hD29 versus staining without 4hD29 (control)) was calculated for Cy5-A and A647-A.

### Nanobody radiolabelling, biodistribution and imaging

His-tagged Nanobodies were radiolabelled with ^99m^Tc by tricarbonyl chemistry^40^ or with ^111^In on DTPA-coupled nanobodies^50^. Prior to *in vivo* use, the radiolabelled tracers were purified by gel-filtration chromatography on Illustra NAP-5 desalting columns (GE Healthcare) and thereafter filtered through a 0.22 µm PVDF membrane filter. All tracers were analysed by instant thin layer chromatography^50,51^ to confirm a radiochemical purity of >95%. The injected ^99m^Tc-tracers 4hD29 or the control BcII10 had an activity of respectively 1.6 ± 0.1 mCi and 1.6 ± 0.1 mCi in the EndoC-βH1 xenograft experiments, and 1.2 ± 0.02 mCi and 1.6 ± 0.1 in Kelly xenograft experiments. ^111^In-labelled Nanobodies were injected at an activity of 6.4 ± 0.2 and 6.9 ± 0.2 µCi for BcII10 and 4hD29, respectively. For SPECT image quantification, 250 mm^3^-sized regions of interest (ROIs) were drawn around each implantation ring on the co-registered SPECT and CT images, and activities in the ROIs were quantified. Mouse anaesthesia, imaging and biodistribution analysis were done as described^40^.

### Statistical analyses

Data are presented as means ± SEM or plotted as scatter or box plots, indicating lower quartile, median, and higher quartile, with whiskers representing the range of the remaining data points. Comparisons were performed by two-tailed paired or unpaired Student’s *t*-test or by ANOVA followed by Student’s *t*-test with Šídák correction, as indicated, using Graph Pad Prism 6 software (Graph Software Inc.). A p-value ≤ 0.05 was considered as statistically significant.

### Data availability

All data generated or analysed during this study are included in this published article and in its Supplementary Information files. The previously published RNAseq dataset analysed during the current study is accessible at GEO: GSE35296.

## Electronic supplementary material

Supplementary info

Supplementary video 1

Supplementary video 2
