# Supplementary material for: A nanobody-based tracer targeting DPP6 for non-invasive imaging of human pancreatic endocrine cells
Source: Sci Rep. 2017 Nov 9;7:15130. doi: 10.1038/s41598-017-15417-2 (PMC5680294; doi:10.1038/s41598-017-15417-2)
Supplement: Supplementary file 1 — Supplementary info [file 41598_2017_15417_MOESM1_ESM.pdf]

A nanobody-based tracer targeting DPP6 for non-invasive imaging of human pancreatic endocrine cells

Alexander Balhuizen[1*], Sam Massa[2,3], Iris Mathijs[4], Jean-Valery Turatsinze[1], Jens De Vos[2,3], Stéphane Demine[1], Catarina Xavier[3], Olatz Villate[1], Isabelle Millard[1], Dominique Egrise[5], Carmen Capito[6], Raphaël Scharfmann[6], Pieter In't Veld[7], Piero Marchetti[8], Serge Muyldermans[3], Serge Goldman[5], Tony Lahoutte[3], Luc Bouwens[4], Decio L. Eizirik[1#*] and Nick Devoogdt[3#]

[1]ULB-Center for Diabetes Research and Welbio, Université Libre de Bruxelles (ULB), Route de Lennik 808-CP618, 1070, Brussels, Belgium.

[2]Laboratory of Cellular and Molecular Immunology (CMIM), Vrije Universiteit Brussel (VUB), Brussels, Belgium.

[3]In vivo Cellular and Molecular Imaging Laboratory (ICMI), Vrije Universiteit Brussel (VUB), Brussels, Belgium.

[4]Cell Differentiation Laboratory, Vrije Universiteit Brussel (VUB), Brussels, Belgium.

[5]Centre for Microscopy and Molecular Imaging (CMMI), Université Libre de Bruxelles (ULB), Gosselies, Belgium.

[6]INSERM U1016, Université Paris-Descartes, Institut Cochin, Paris, France.

[7]Department of Pathology, UZ-Brussel, Vrije Universiteit Brussel (VUB), Brussels, Belgium.

[8]Department of Endocrinology and Metabolism, University of Pisa, Pisa, Italy.

Supplementary Table 1: Basic clinical characteristics of the organ donors used for human islet isolation or pancreas histology.

Sample identity	Gender	Age (years)	BMI (Kg/m^2)	Purity (% beta cells)	T1D (years)	Cause of Death
**qPCR**						
ID210509	Female	79	28.1	61	-	Trauma
ID291108	Female	77	23.8	45	-	Trauma
ID151214	Male	79	25.3	48	-	Cerebral hemorrhage
ID110215	Male	44	27.7	59	-	Post-anoxic encephalopathy
*ID230217	Male	85	24.2	-	-	Cerebral hemorrhage
*ID240217	Female	82	26.1	-	-	Post-anoxic encephalopathy
*ID130317	Male	71	22	-	-	Trauma
*ID2010_1						
*ID2010_2						
*ID2010_3						
**IHC**						
HP3540	Female	66	-	-	19	Intracerebral bleeding
HP817	Male	32	-	-	16, IAA,IA2Ab+	Intracerebral bleeding
A01/124	Female	27	-	-	Unknown, “chronic”	Intracerebral bleeding
HP2147	Male	33	24.5	-	-	Fact. cranii, cont. cerebra
HP2875	Female	23	22	-	-	Cerebral edema
HP3460	Female	62	21	-	-	Trauma
**FC**						
HP3442	Female	52	20.2	-	-	Intracerebral bleeding
HP3444	Male	48	26.1	-	-	Unspecified
ID230217	Male	85	24.2	-	-	Cerebral hemorrhage
ID240217	Female	82	26.1	-	-	Post-anoxic encephalopathy

Whole pancreata were used for histology with immunohistochemistry (IHC), while human isolated islets were used for quantitative PCR (qPCR), and digested pancreata were used for flow cytometry (FC). An asterisk indicates the exocrine material used for qPCR, collected after pancreatic islet removal. All donors were anonymized and the material was annotated with a sample identity.

Supplementary figure 1

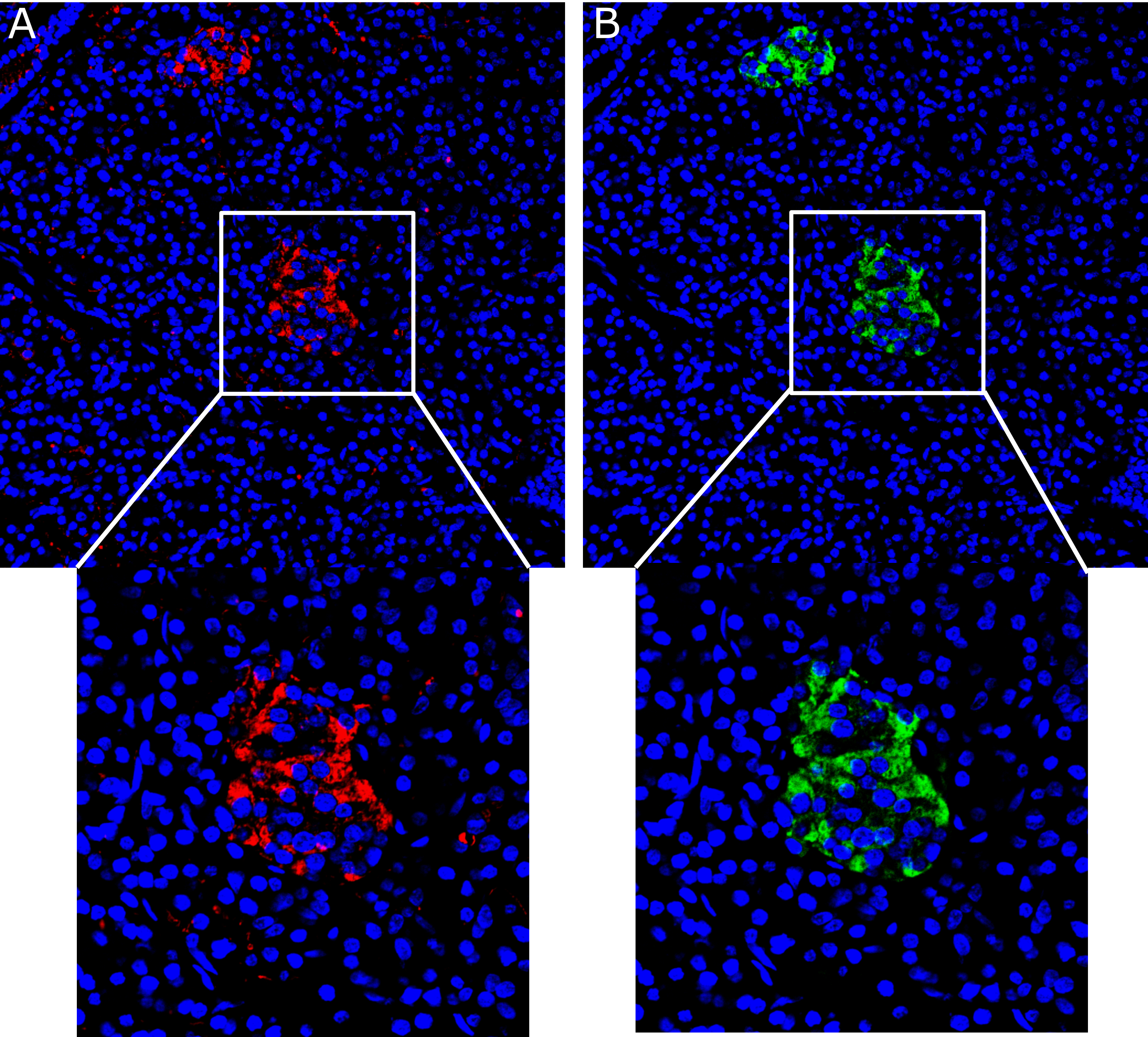

Supplementary figure 2

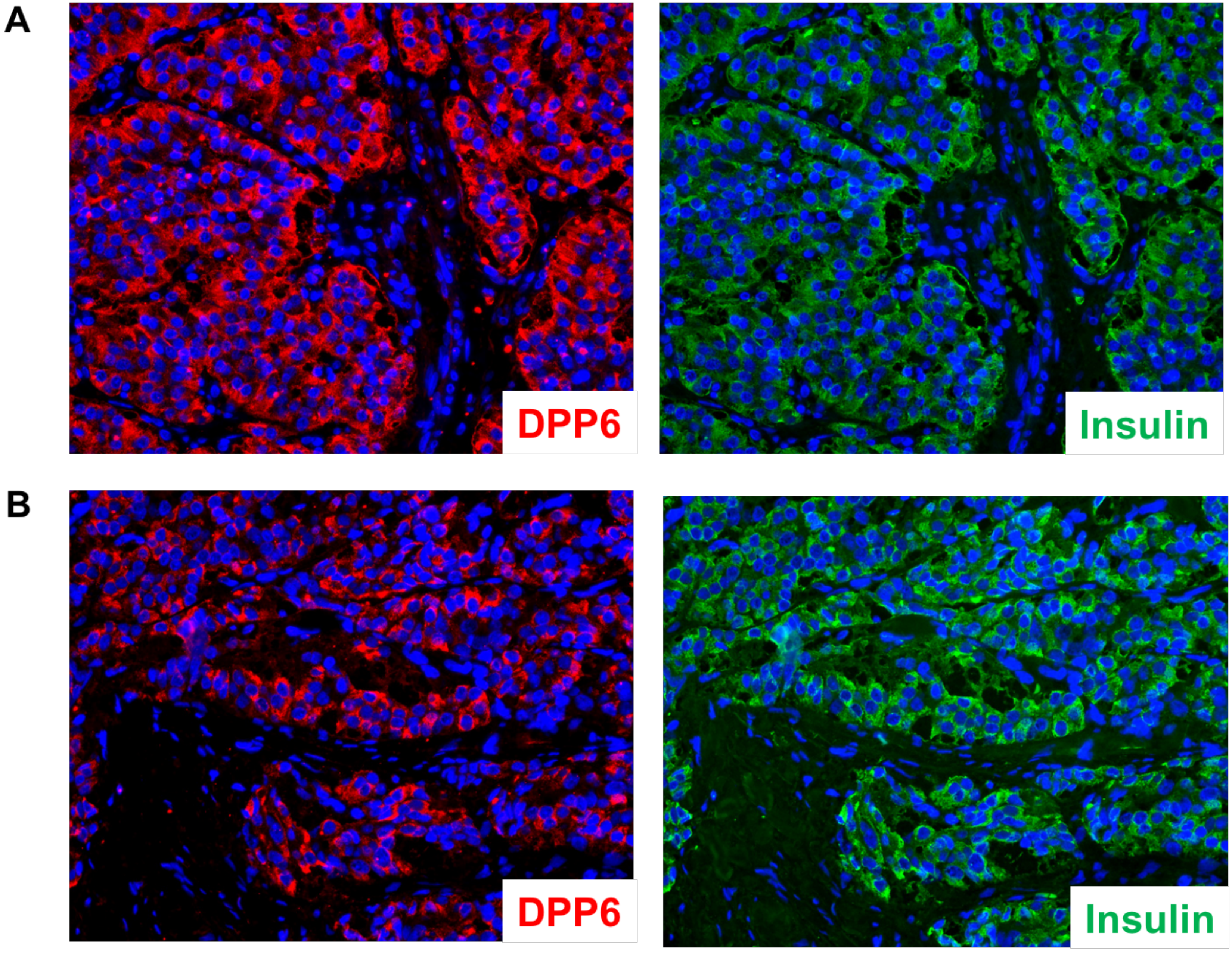

Supplementary figure 3

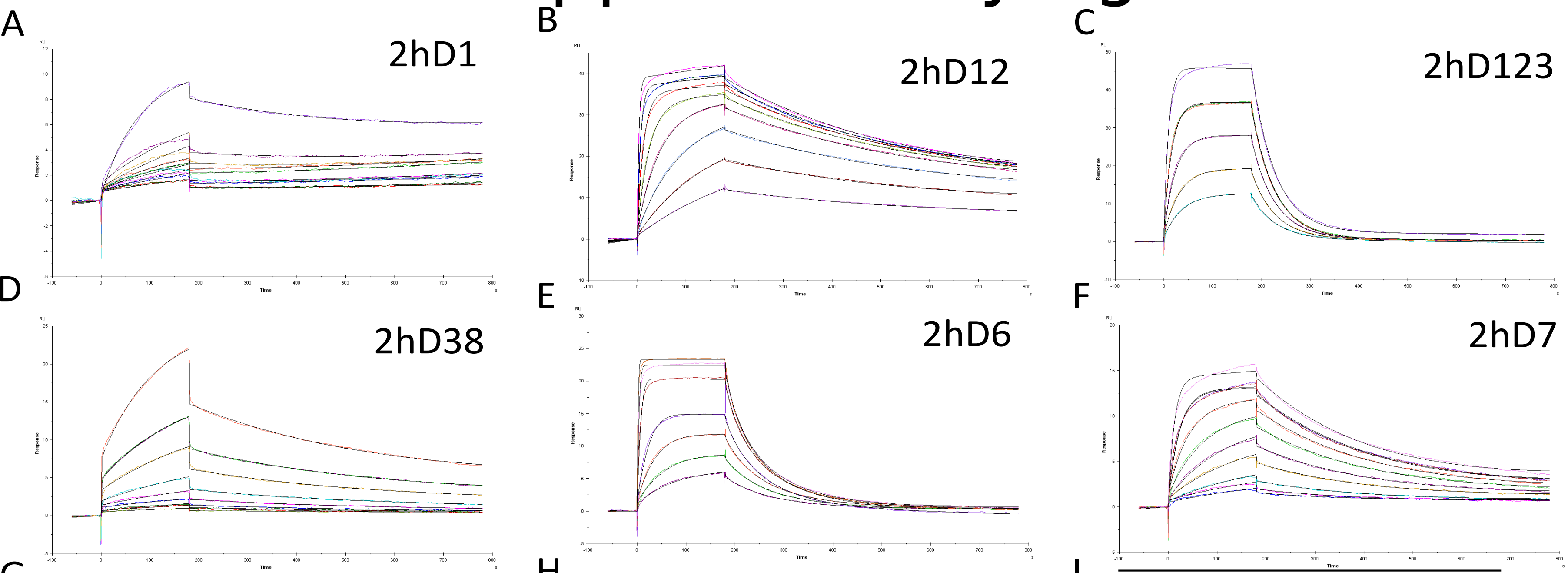

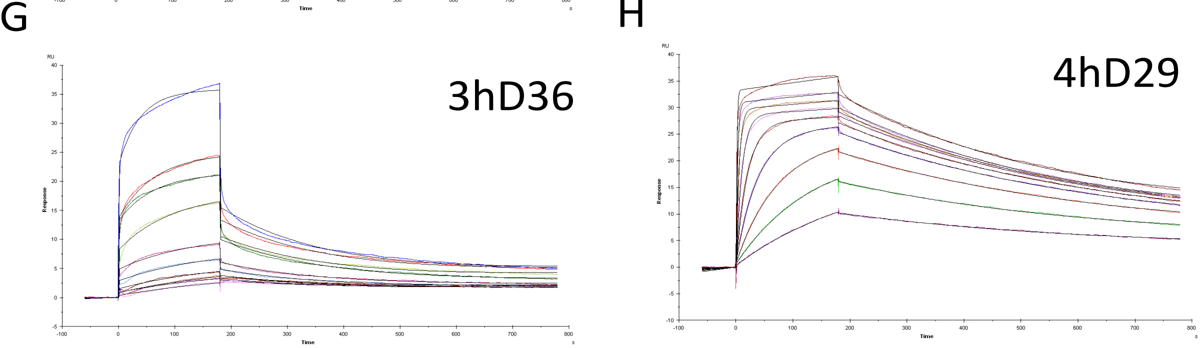

I

Protein	Affinity (K_D, nmol/l)
2hD1	69
2hD12	3.1
2hD123	200
2hD38	145
2hD6	13
2hD7	25
3hD36	31
4hD29	1.2

J

```
       1        10        20        30        40        50        60        70        80        90        100       115       120
        ******** ********* ****** *** ******** * ********* ***** **** ***** **** ********* ********* ********* **** ***** *1234554321****** ** ********
       <-------FR1-IMGT--------> <-CDR1-IMGT> <---FR2-IMGT----> <CDR2IMGT> <--------------FR3-IMGT----------------> <-------CDR3-IMGT-----> <FR4-IMGT->
2hD1   QVQLQESGG-GLVQPGGSLRLSCAAS GYPYGYTFSSYC MRWFRQAPGKDREGVAR FERN--GLTT YYDDSVK-GRFTISQDNVKNTVYLQMNSLKPEDTATYYC AAAPKQLR--------TCGDYNY WGQGTQVTVSS
2hD123 QVQLQESGG-GSVQAGGSLRLSCAAS GSSY----SRFR MGWFRQVPGKEREGVAA IYRS--DGRT YYADSVK-GRFTISQDNTKNTVYLQMNSLKPEDTAMYYC AAGAYSSY-------LMDANFAY WGQGTQVTVSS
2hD38  QVQLQESGG-GSVQAGGSLRLSCAAS ENTQ----GNYC LAWFRQAPGKEREGVAS ISSG--GIKT YYADTVK-GRFTISRDNAENTVYLQMNSLKPEDTAIYYC AARTSVTCFAS--SWARLNAYAY WGQGTQVTVSS
2hD6   QVQLQESGG-GSVQAGGSLRLSCAAS SYTY----SYSC MAWFRQAPGKERERVAS IHTG--TGTA NYADSAK-GRFTISQDIAANTVYLQMNSLKLEDTAMYYC AARPGSAALRCTTDYSKPHDFTY WGQGTQVTVSS
4hD29  QVQLQESGG-GLVQPGGSLRLSCAAS GFTF----SSNY MTWVRQAPGKGPEWVSG INPD--GSST YYADSVK-GRFTISRDNAKNTLYLQMNSLKSEDTALYKC ATGAAP-----------RIPTTL RGQGTQVTVSS
```

Supplementary figure 4

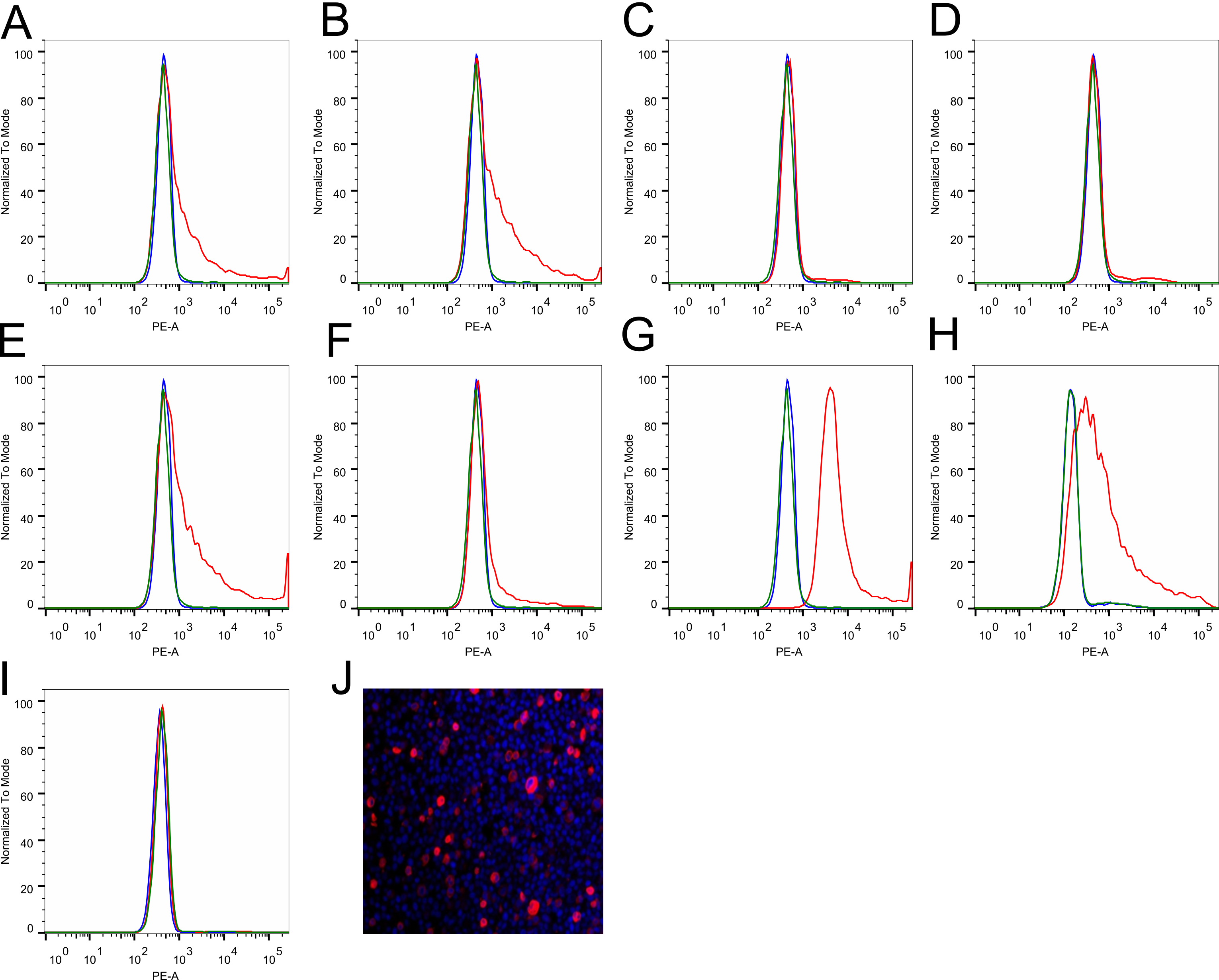

Supplementary figure 5

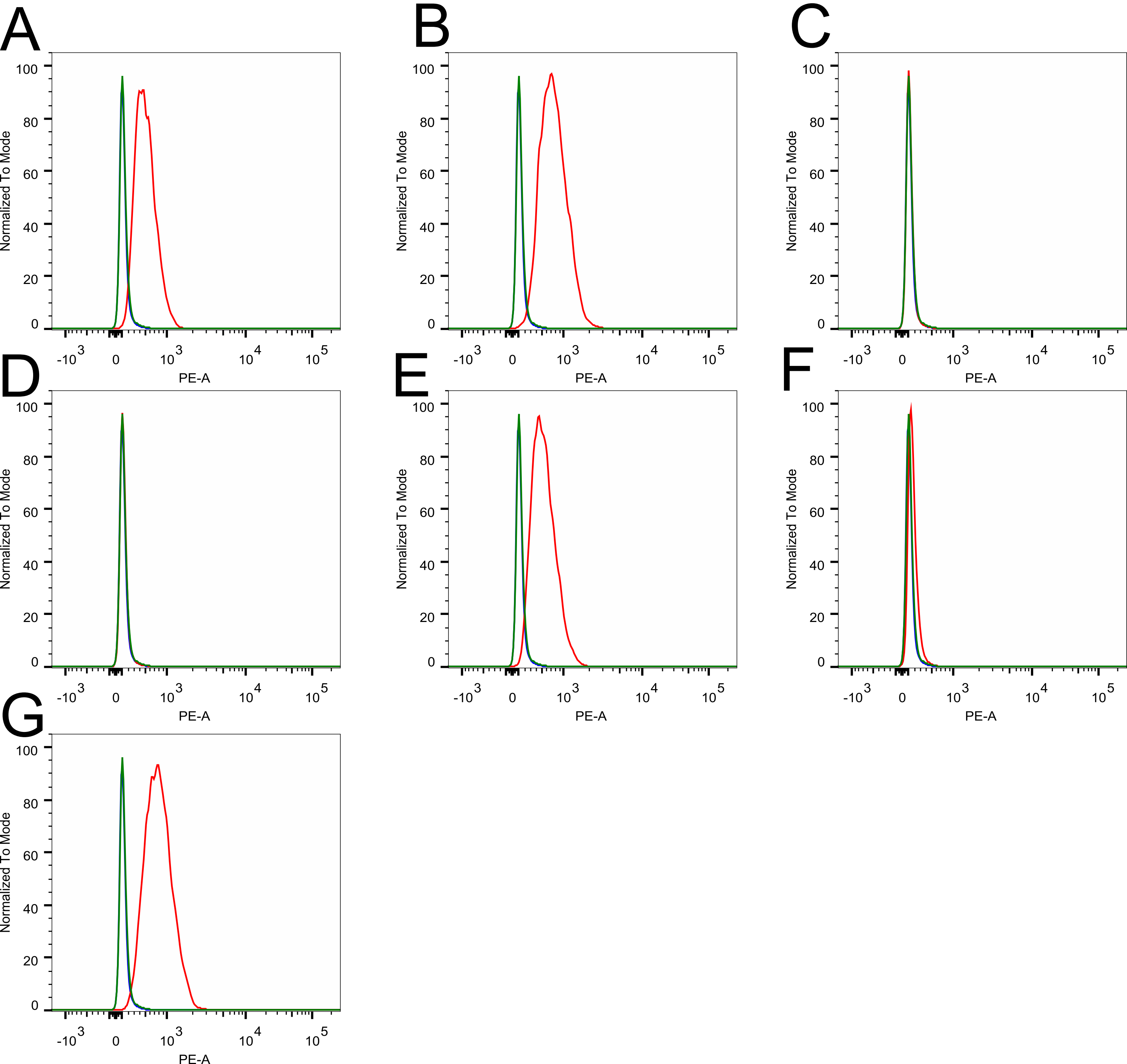

Supplementary figure 6

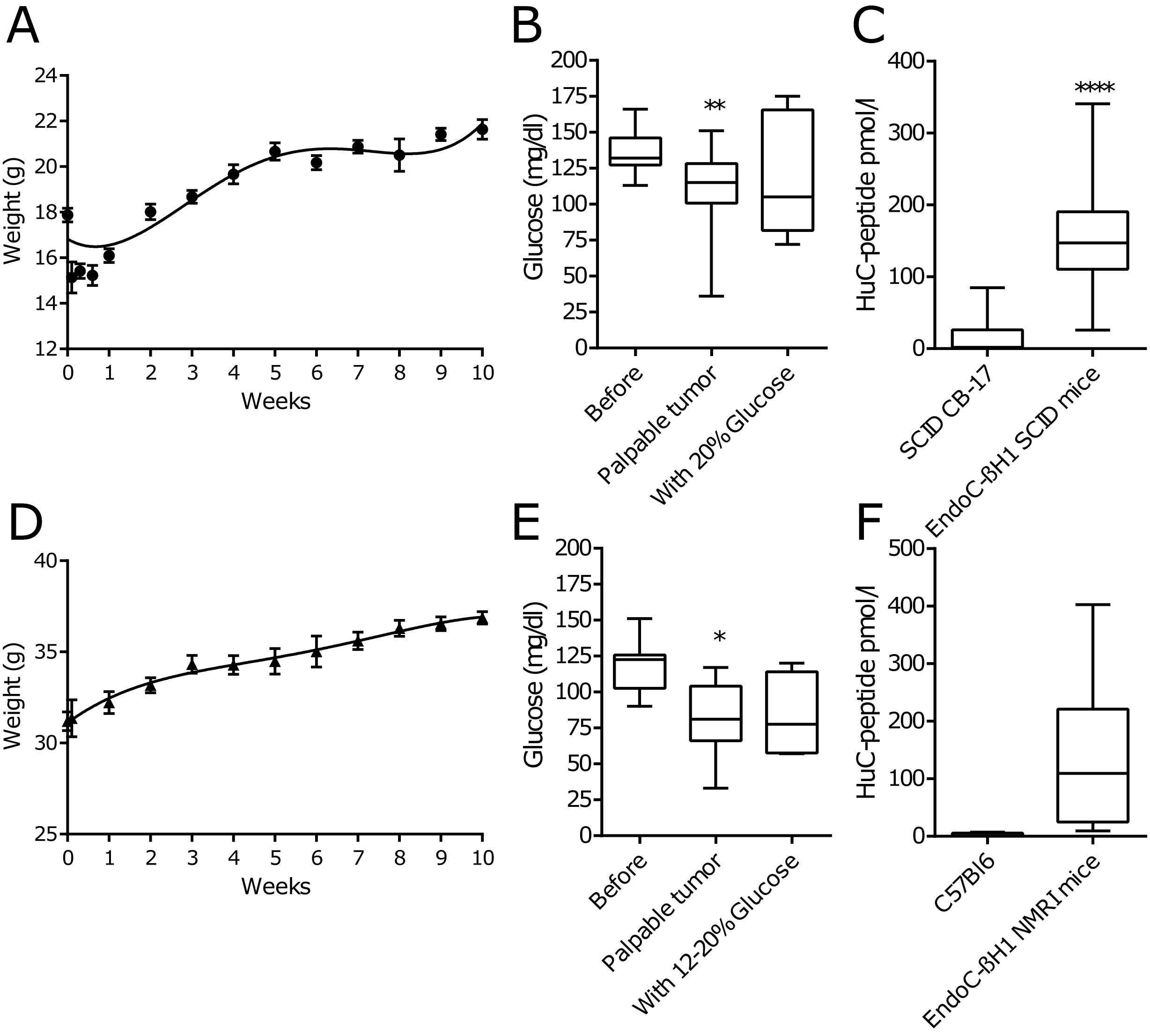

Supplementary figure 7

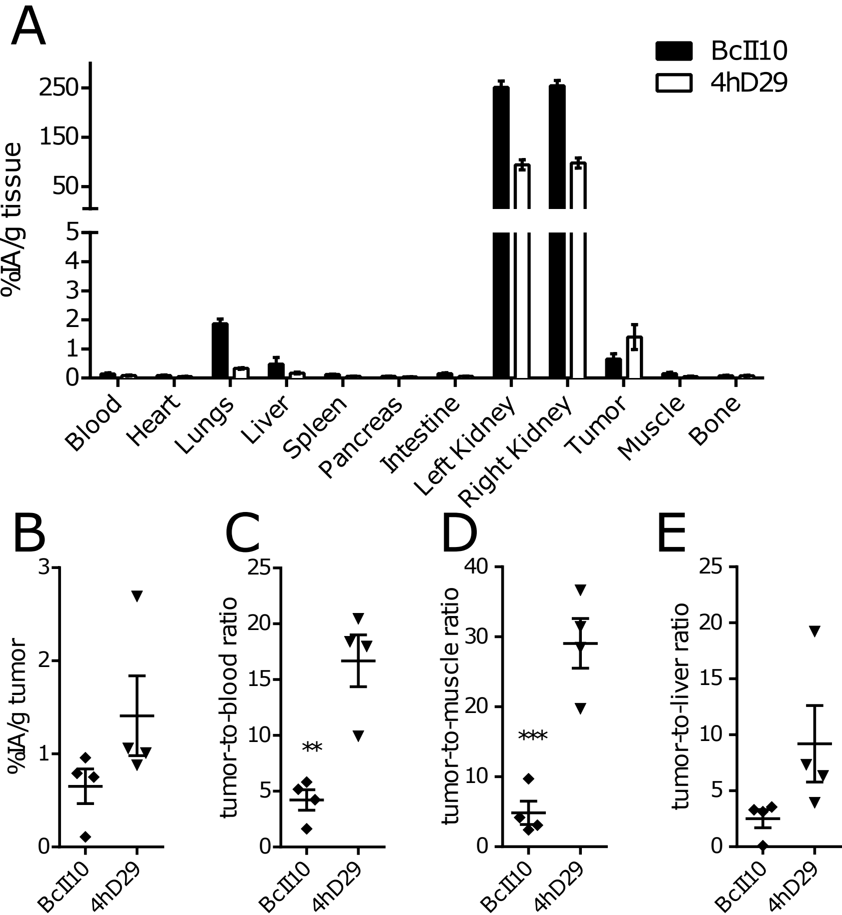

Supplementary figure 8

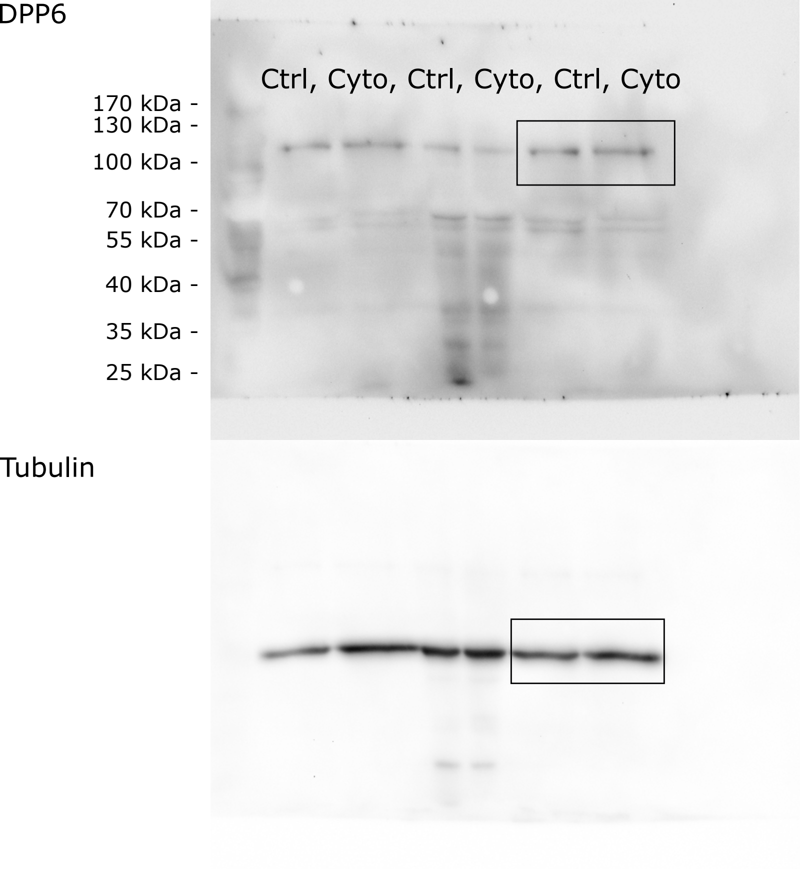

Supplementary figure legends

Supplementary Figure 1. Preferential localization of DPP6 in the endocrine part of the pancreas.

Immunohistochemistry was used to determine the localization of DPP6 in the human pancreas. (A) DPP6 is colored in red and DAPI, indicating nuclear staining, in blue; (B) insulin is colored in green and DAPI in blue. The figure shown is representative of 3 preparations.

Supplementary Figure 2. Insulinomas express DPP6.

A. Insulinoma grade 2 of 1.9cm from a 42-year old patient, located in the tail part of the pancreas. The tumoural cells are positive for insulin (green) and DPP6 (red). There is clear superposition between insulin and DPP+ cells; B. Insulinoma grade 2 of 4.1cm from a 31-year female patient, located in the uncinate part of the pancreas. DPP6 is stained red and insulin green (same antibodies as in Figure 4). There is again a clear superposition between insulin and DPP+ cells.

Supplementary Figure 3. The affinity of the candidate nanobodies for the DPP6 protein.

Surface plasmon resonance (SPR) was used to evaluate the affinity of the top eight nanobody candidates for the extracellular part of DPP6. Nine different dilutions (2 – 500 nmol/l) of the nanobodies reacted against immobilized recombinant human DPP6 and both K_{on}-rate and K_{off}-rates were monitored and quantified. (A) 2hD1; (B) 2hD12; (C) 2hD123; (D) 2hD38; (E) 2hD6; (F) 2hD7; (G) 3hD36; (H) 4hD29; (I) Surface plasmon resonance (SPR) affinities (K_D values); (J) Amino acid sequence of the best five anti-DPP6 nanobodies.

Supplementary Figure 4. Flow cytometry analysis using CHO cells overexpressing human DPP6 to validate the selected nanobodies.

Flow cytometry analysis of CHO cells transiently transfected with human DPP6 (DPP6+CHO) and then labeled with 9 different nanobody candidates (red line) to evaluate their binding to these cells: (A) 2hD1; (B) 2hD6; (C) 2hD7; (D) 2hD12; (E) 2hD38; (F) 2hD123; (G) 3hD36; (H) 4hD29. In addition, (I) CHO cells transfected with the murine DPP6 orthologue were also labeled with 4hD29. As controls, the cells were labeled with an irrelevant control nanobody (green) or omitting Nb (blue, anti-His monoclonal antibody (mAb) and anti-mouse-IgG-PE detecting antibodies only). (J) Immunocytochemistry picture of the human DPP6+CHO cells stained with an anti-human DPP6 mAb, indicating an estimated transfection efficiency of 20-40%. A-J are representative images for n=1 for all candidates except for 4hD29, where representative data is shown from n=3.

Supplementary Figure 5. Flow cytometry analysis using Kelly Neuroblastoma cells to validate the selected nanobodies.

Flow cytometry analysis of Kelly neuroblastoma cells labeled with 7 different Nanobody candidates (red line) to evaluate their affinity to these cells: (A) 2hD1; (B) 2hD6; (C) 2hD7; (D) 2hD12; (E) 2hD38; (F) 2hD123; (G) 4hD29. As controls, the cells were labeled with an irrelevant control nanobody (green) or omitting Nanobody (blue, anti-His monoclonal antibody (mAb) and anti-mouse-IgG-PE detecting antibodies only). N=1 for all candidates except for 4hD29, where representative data is shown from n=4.

Supplementary Figure 6. Evolution of body weight, glucose and C-peptide blood concentrations in NMRI-*Foxn1$^{nu/}$Foxn1^{nu}* (NMRI-EndoC) and SCID-CB-17/Icr-*Prkdc^{scid}*/Rj (SCID-EndoC)-EndoC-βH1 grafted mice.
Immunodeficient SCID mice (A, n=24) or NMRI mice (D, n=8) implanted with human EndoC-βH1 cells gained weight, as expected. (B) Random glycemic levels were determined in the SCID-EndoC mice before tumor implantation, when the tumor became palpable (after 9-10 weeks) and after the mice received glucose-supplemented water (n=10-24). (C) In parallel to the last glycemic measurements, human C-peptide was determined in SCID-EndoC mice (n=10), and compared to the initial control values (SCID CB-17). (E-F) Random glycemic levels and (F) human C-peptide were also determined as above in NMRI-EndoC mice, (n=4-8) with C57Bl6 mice (n=4) used as an external control. One way ANOVA with Šídák correction for multiple comparisons were performed in panels B and E to compare 3 groups, while in panel C unpaired Student's *t*-test was performed to compare two groups; *p≤0.05, **p≤0.01, ****p≤0.0001.

Supplementary Figure 7. *Ex vivo* binding pattern of the ^{111}In-4hD29 tracer.
Ex vivo biodistribution of the ^{111}In-4hD29 or control Nanobody (Nb) Bcll10 tracers in mice bearing a Kelly neuroblastoma tumor (n=4). The dissected tissues were collected 2h post injection and quantified by gamma-counting. (A) Quantification plot of 12 tissues expressed as percentage injected activity per gram of tissue (%IA/g); (B) Quantification of the tumor containing ^{111}In-4hD29 or control ^{111}In-BcII10, expressed in %IA/g; (C) Tumor-to-blood ratio of ^{111}In-4hD29 and ^{111}In-BcII10; (D) Tumor-to-muscle ratio of ^{111}In-4hD29 and the control tracer ^{111}In-BcII10. Data are means ± SEM; n=4; unpaired *t*-test, **p≤0.01; ***p≤0.001.

Supplementary Figure 8. Supplementary full length blots for cropped blots for DPP6 (black box) and Tubulin shown in figure 3B for control and cytokine treated EndoC-βH1 cells.

Supplementary Videos 1 and 2. 3D rendered overview of merged SPECT/CT image of EndoC-βH1-mice.
Representative 3D rendered movies of a SCID mouse bearing an EndoC-βH1 tumor (Video 1) in its hind leg from a merged SPECT-CT *in vivo* image with the ^{99m}Tc-4hD29 tracer; (n=4); an inactive control nanobody was also injected in the same mouse model (Video 2). The ring placed on the right contains EndoC-βH1 cells, while the vehicle ring is located on the left side.
